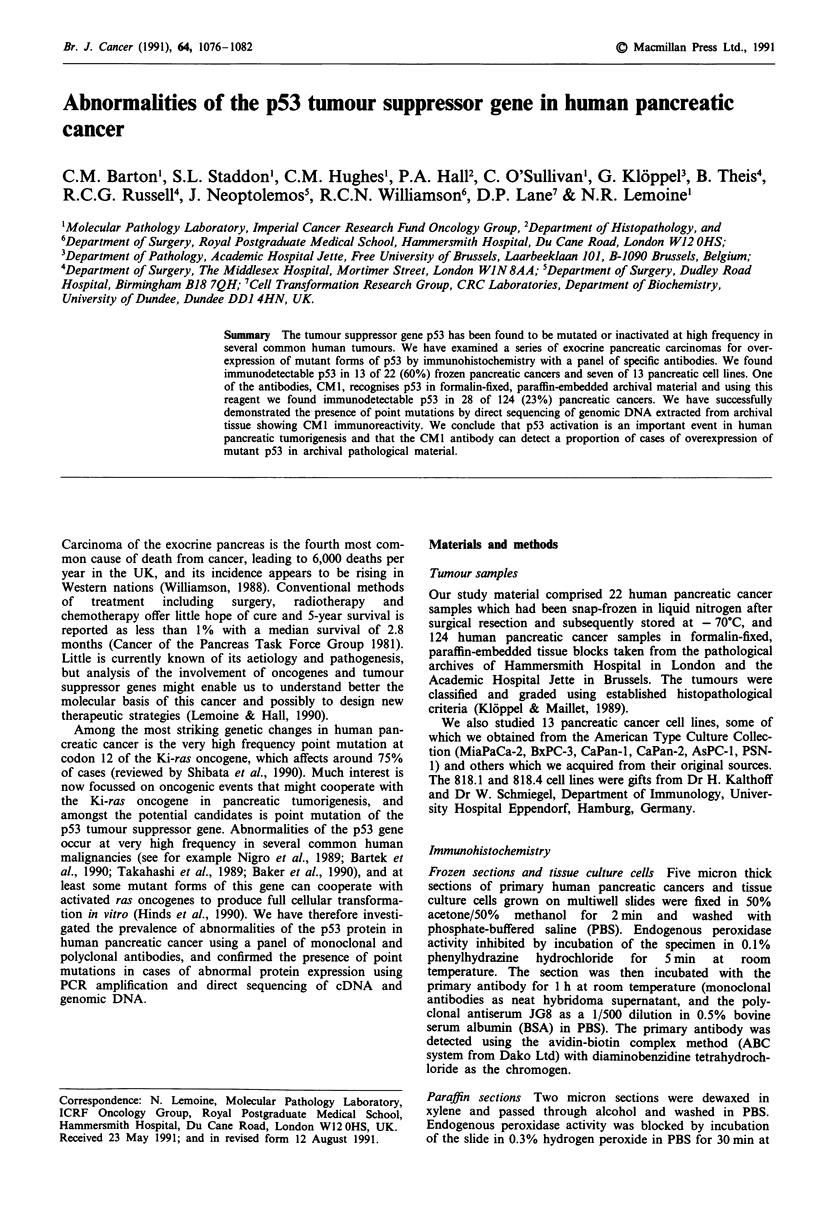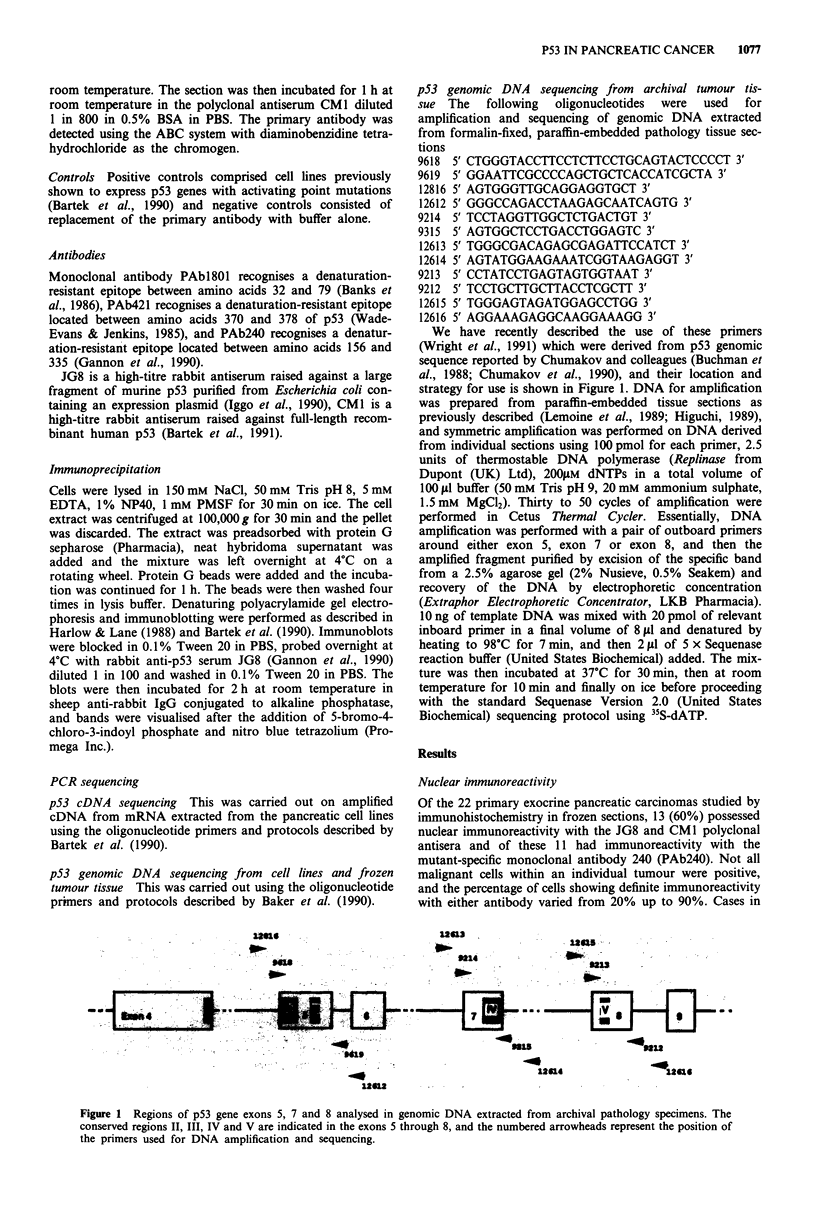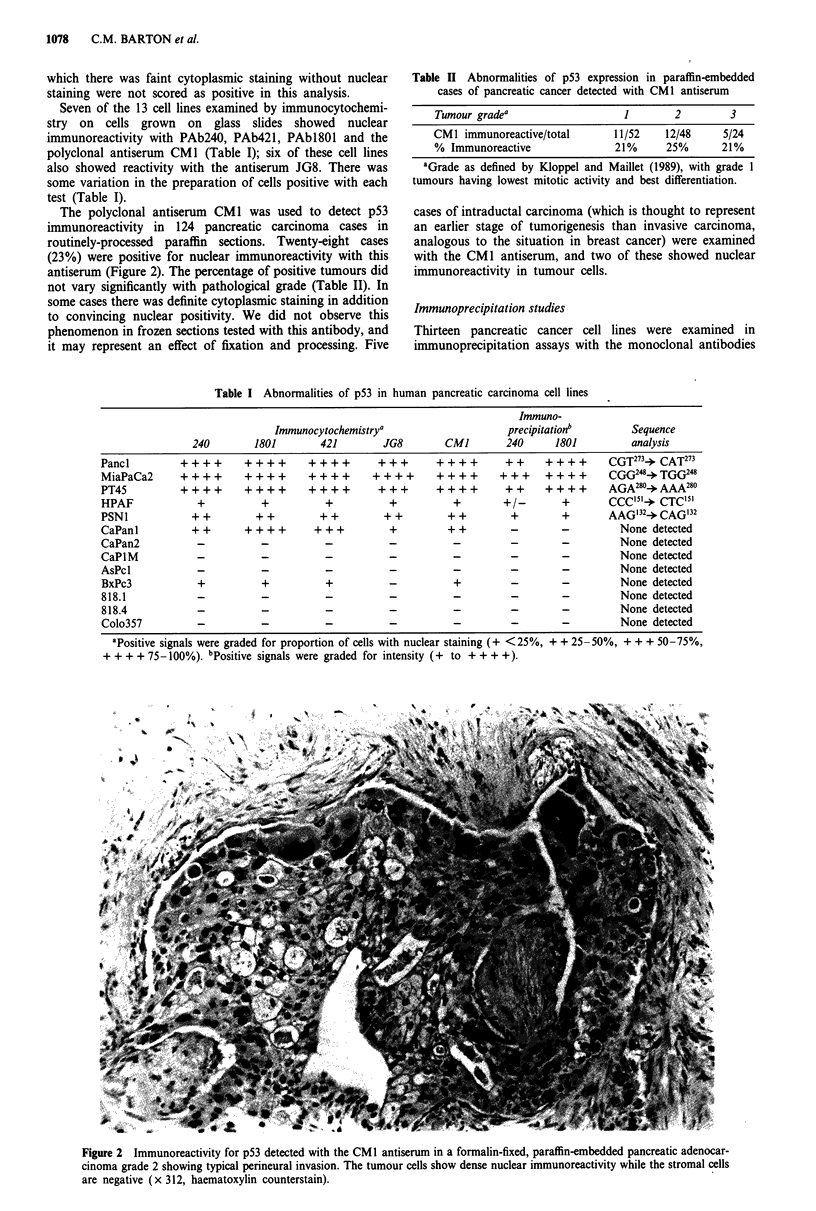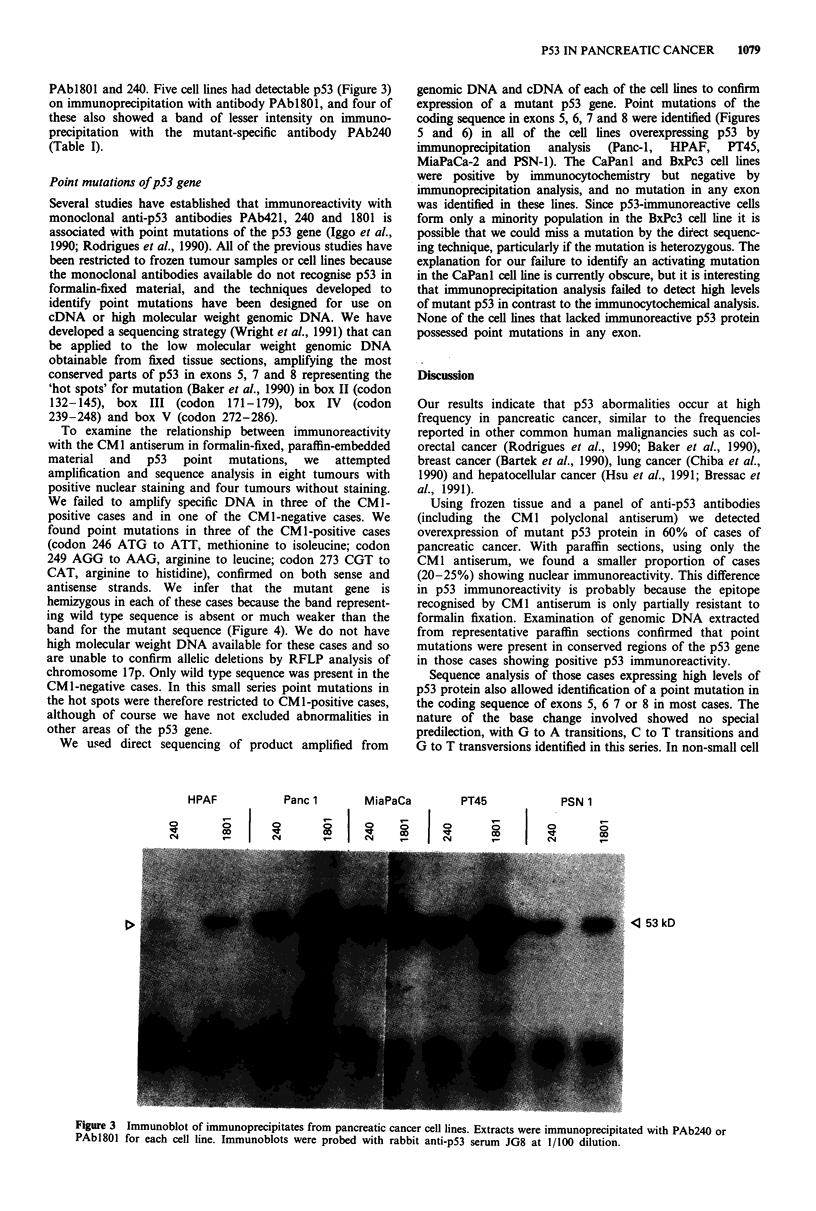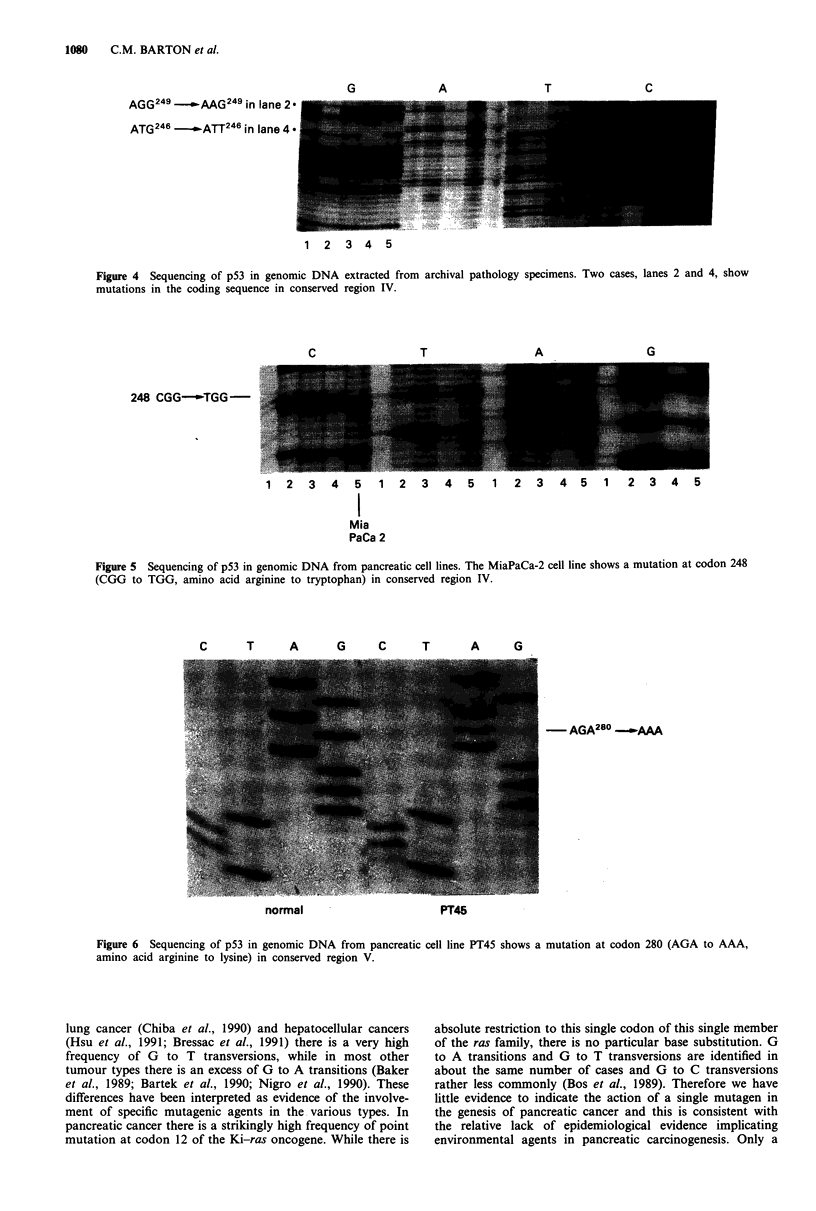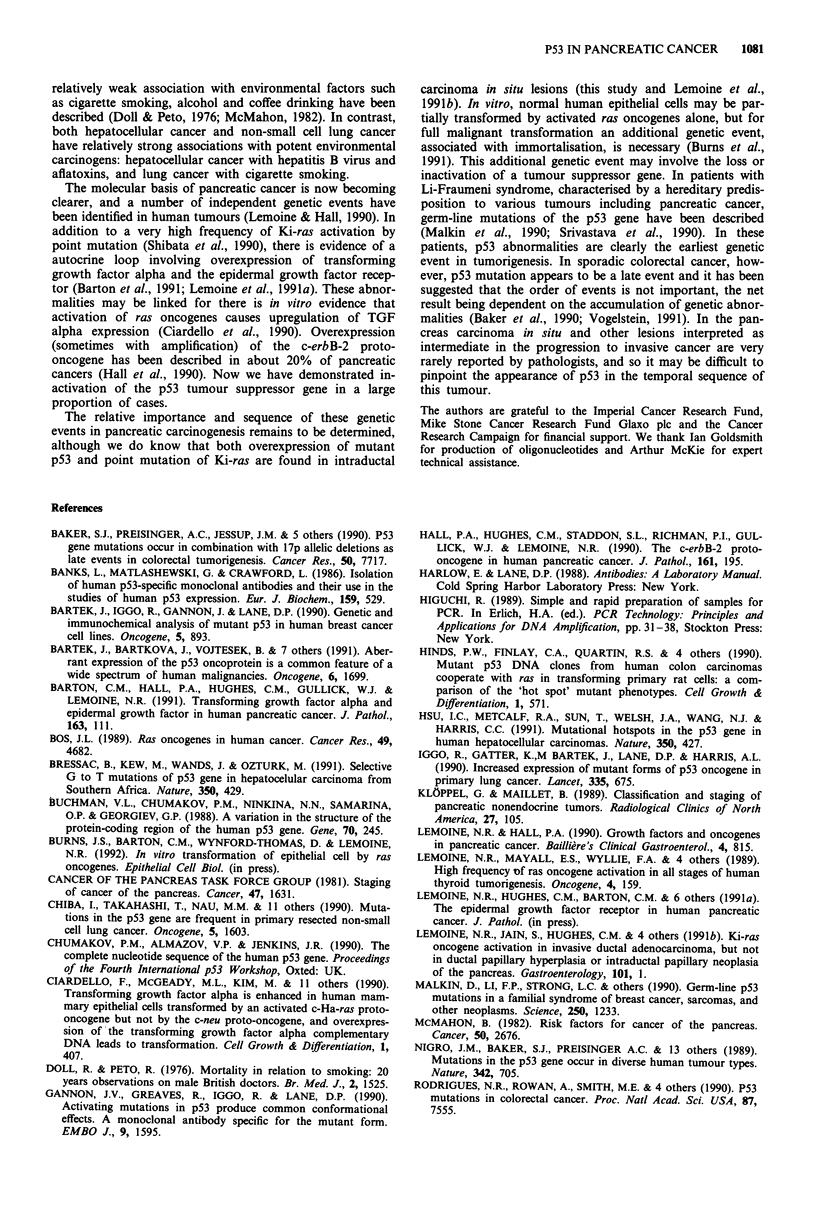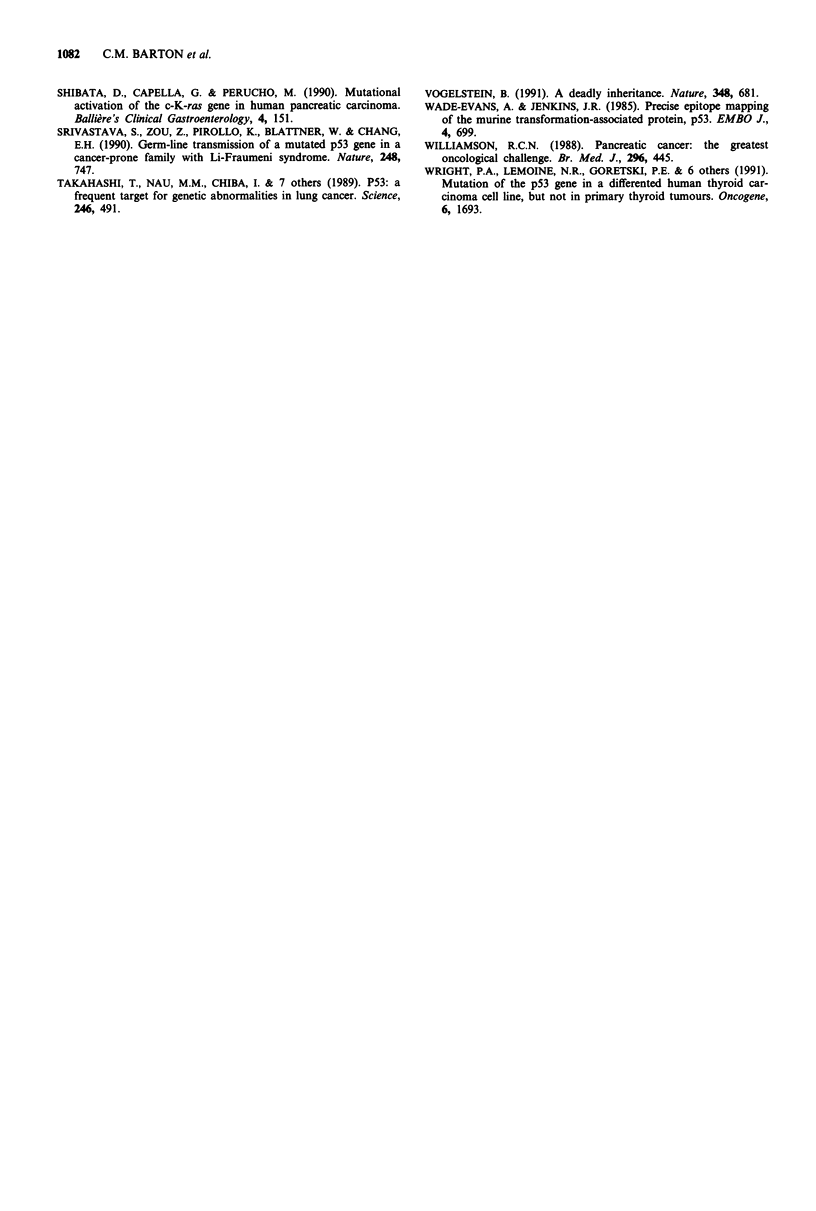# Abnormalities of the p53 tumour suppressor gene in human pancreatic cancer.

**DOI:** 10.1038/bjc.1991.467

**Published:** 1991-12

**Authors:** C. M. Barton, S. L. Staddon, C. M. Hughes, P. A. Hall, C. O'Sullivan, G. Klöppel, B. Theis, R. C. Russell, J. Neoptolemos, R. C. Williamson

**Affiliations:** Molecular Pathology Laboratory, Imperial Cancer Research Fund Oncology Group, London, UK.

## Abstract

**Images:**


*Br. J. Cancer* (1991), **64**, 1076–1082 © Macmillan Press Ltd., 1991

### Abnormalities of the p53 tumour suppressor gene in human pancreatic cancer

C.M. Barton[1], S.L. Staddon[1], C.M. Hughes[1], P.A. Hall[2], C. O'Sullivan[1], G. Klöppel[3], B. Theis[4], R.C.G. Russell[4], J. Neoptolemos[5], R.C.N. Williamson[6], D.P. Lane[7] & N.R. Lemoine[1]

[1]*Molecular Pathology Laboratory, Imperial Cancer Research Fund Oncology Group,* [2]*Department of Histopathology, and* [6]*Department of Surgery, Royal Postgraduate Medical School, Hammersmith Hospital, Du Cane Road, London W12 0HS;* [3]*Department of Pathology, Academic Hospital Jette, Free University of Brussels, Laarbeeklaan 101, B-1090 Brussels, Belgium;* [4]*Department of Surgery, The Middlesex Hospital, Mortimer Street, London W1N 8AA;* [5]*Department of Surgery, Dudley Road Hospital, Birmingham B18 7QH;* [7]*Cell Transformation Research Group, CRC Laboratories, Department of Biochemistry, University of Dundee, Dundee DD1 4HN, UK.*

**Summary** The tumour suppressor gene p53 has been found to be mutated or inactivated at high frequency in several common human tumours. We have examined a series of exocrine pancreatic carcinomas for overexpression of mutant forms of p53 by immunohistochemistry with a panel of specific antibodies. We found immunodetectable p53 in 13 of 22 (60%) frozen pancreatic cancers and seven of 13 pancreatic cell lines. One of the antibodies, CM1, recognises p53 in formalin-fixed, paraffin-embedded archival material and using this reagent we found immunodetectable p53 in 28 of 124 (23%) pancreatic cancers. We have successfully demonstrated the presence of point mutations by direct sequencing of genomic DNA extracted from archival tissue showing CM1 immunoreactivity. We conclude that p53 activation is an important event in human pancreatic tumorigenesis and that the CM1 antibody can detect a proportion of cases of overexpression of mutant p53 in archival pathological material.

Carcinoma of the exocrine pancreas is the fourth most common cause of death from cancer, leading to 6,000 deaths per year in the UK, and its incidence appears to be rising in Western nations (Williamson, 1988). Conventional methods of treatment including surgery, radiotherapy and chemotherapy offer little hope of cure and 5-year survival is reported as less than 1% with a median survival of 2.8 months (Cancer of the Pancreas Task Force Group 1981). Little is currently known of its aetiology and pathogenesis, but analysis of the involvement of oncogenes and tumour suppressor genes might enable us to understand better the molecular basis of this cancer and possibly to design new therapeutic strategies (Lemoine & Hall, 1990).

Among the most striking genetic changes in human pancreatic cancer is the very high frequency point mutation at codon 12 of the Ki-*ras* oncogene, which affects around 75% of cases (reviewed by Shibata *et al.*, 1990). Much interest is now focussed on oncogenic events that might cooperate with the Ki-*ras* oncogene in pancreatic tumorigenesis, and amongst the potential candidates is point mutation of the p53 tumour suppressor gene. Abnormalities of the p53 gene occur at very high frequency in several common human malignancies (see for example Nigro *et al.*, 1989; Bartek *et al.*, 1990; Takahashi *et al.*, 1989; Baker *et al.*, 1990), and at least some mutant forms of this gene can cooperate with activated *ras* oncogenes to produce full cellular transformation *in vitro* (Hinds *et al.*, 1990). We have therefore investigated the prevalence of abnormalities of the p53 protein in human pancreatic cancer using a panel of monoclonal and polyclonal antibodies, and confirmed the presence of point mutations in cases of abnormal protein expression using PCR amplification and direct sequencing of cDNA and genomic DNA.

Correspondence: N. Lemoine, Molecular Pathology Laboratory, ICRF Oncology Group, Royal Postgraduate Medical School, Hammersmith Hospital, Du Cane Road, London W12 0HS, UK.
Received 23 May 1991; and in revised form 12 August 1991.

### Materials and methods

### Tumour samples

Our study material comprised 22 human pancreatic cancer samples which had been snap-frozen in liquid nitrogen after surgical resection and subsequently stored at − 70°C, and 124 human pancreatic cancer samples in formalin-fixed, paraffin-embedded tissue blocks taken from the pathological archives of Hammersmith Hospital in London and the Academic Hospital Jette in Brussels. The tumours were classified and graded using established histopathological criteria (Klöppel & Maillet, 1989).

We also studied 13 pancreatic cancer cell lines, some of which we obtained from the American Type Culture Collection (MiaPaCa-2, BxPC-3, CaPan-1, CaPan-2, AsPC-1, PSN-1) and others which we acquired from their original sources. The 818.1 and 818.4 cell lines were gifts from Dr H. Kalthoff and Dr W. Schmiegel, Department of Immunology, University Hospital Eppendorf, Hamburg, Germany.

### Immunohistochemistry

*Frozen sections and tissue culture cells* Five micron thick sections of primary human pancreatic cancers and tissue culture cells grown on multiwell slides were fixed in 50% acetone/50% methanol for 2 min and washed with phosphate-buffered saline (PBS). Endogenous peroxidase activity inhibited by incubation of the specimen in 0.1% phenylhydrazine hydrochloride for 5 min at room temperature. The section was then incubated with the primary antibody for 1 h at room temperature (monoclonal antibodies as neat hybridoma supernatant, and the polyclonal antiserum JG8 as a 1/500 dilution in 0.5% bovine serum albumin (BSA) in PBS). The primary antibody was detected using the avidin-biotin complex method (ABC system from Dako Ltd) with diaminobenzidine tetrahydrochloride as the chromogen.

*Paraffin sections* Two micron sections were dewaxed in xylene and passed through alcohol and washed in PBS. Endogenous peroxidase activity was blocked by incubation of the slide in 0.3% hydrogen peroxide in PBS for 30 min at room temperature. The section was then incubated for 1 h at room temperature in the polyclonal antiserum CM1 diluted 1 in 800 in 0.5% BSA in PBS. The primary antibody was detected using the ABC system with diaminobenzidine tetrahydrochloride as the chromogen.

*Controls* Positive controls comprised cell lines previously shown to express p53 genes with activating point mutations (Bartek *et al.*, 1990) and negative controls consisted of replacement of the primary antibody with buffer alone.

### *Antibodies*

Monoclonal antibody PAb1801 recognises a denaturation-resistant epitope between amino acids 32 and 79 (Banks *et al.*, 1986), PAb421 recognises a denaturation-resistant epitope located between amino acids 370 and 378 of p53 (Wade-Evans & Jenkins, 1985), and PAb240 recognises a denaturation-resistant epitope located between amino acids 156 and 335 (Gannon *et al.*, 1990).

JG8 is a high-titre rabbit antiserum raised against a large fragment of murine p53 purified from *Escherichia coli* containing an expression plasmid (Iggo *et al.*, 1990), CM1 is a high-titre rabbit antiserum raised against full-length recombinant human p53 (Bartek *et al.*, 1991).

### *Immunoprecipitation*

Cells were lysed in 150 mM NaCl, 50 mM Tris pH 8, 5 mM EDTA, 1% NP40, 1 mM PMSF for 30 min on ice. The cell extract was centrifuged at 100,000 *g* for 30 min and the pellet was discarded. The extract was preadsorbed with protein G sepharose (Pharmacia), neat hybridoma supernatant was added and the mixture was left overnight at 4°C on a rotating wheel. Protein G beads were added and the incubation was continued for 1 h. The beads were then washed four times in lysis buffer. Denaturing polyacrylamide gel electrophoresis and immunoblotting were performed as described in Harlow & Lane (1988) and Bartek *et al.* (1990). Immunoblots were blocked in 0.1% Tween 20 in PBS, probed overnight at 4°C with rabbit anti-p53 serum JG8 (Gannon *et al.*, 1990) diluted 1 in 100 and washed in 0.1% Tween 20 in PBS. The blots were then incubated for 2 h at room temperature in sheep anti-rabbit IgG conjugated to alkaline phosphatase, and bands were visualised after the addition of 5-bromo-4-chloro-3-indoyl phosphate and nitro blue tetrazolium (Promega Inc.).

### *PCR sequencing*

*p53 cDNA sequencing* This was carried out on amplified cDNA from mRNA extracted from the pancreatic cell lines using the oligonucleotide primers and protocols described by Bartek *et al.* (1990).

*p53 genomic DNA sequencing from cell lines and frozen tumour tissue* This was carried out using the oligonucleotide primers and protocols described by Baker *et al.* (1990).

*p53 genomic DNA sequencing from archival tumour tissue* The following oligonucleotides were used for amplification and sequencing of genomic DNA extracted from formalin-fixed, paraffin-embedded pathology tissue sections

9618 5' CTGGGTACCTTCCTCTTCCTGCAGTACTCCCCT 3'
9619 5' GGAATTCGCCCCAGCTGCTCACCATCGCTA 3'
12816 5' AGTGGGTT.GCAGGAGGTGCT 3'
12612 5' GGGCCAGACCTAAGAGCAATCAGTG 3'
9214 5' TCCTAGGTTGGCTCTGACTGT 3'
9315 5' AGTGGCTCCTGACCTGGAGTC 3'
12613 5' TGGGCGACAGAGCGAGATTCCATCT 3'
12614 5' AGTATGGAAGAAATCGGTAAGAGGT 3'
9213 5' CCTATCCTGAGTAGTGGTAAT 3'
9212 5' TCCTGCTTGCTTACCTCGCTT 3'
12615 5' TGGGAGTAGATGGAGCCTGG 3'
12616 5' AGGAAAGAGGCAAGGAAAGG 3'

We have recently described the use of these primers (Wright *et al.*, 1991) which were derived from p53 genomic sequence reported by Chumakov and colleagues (Buchman *et al.*, 1988; Chumakov *et al.*, 1990), and their location and strategy for use is shown in Figure 1. DNA for amplification was prepared from paraffin-embedded tissue sections as previously described (Lemoine *et al.*, 1989; Higuchi, 1989), and symmetric amplification was performed on DNA derived from individual sections using 100 pmol for each primer, 2.5 units of thermostable DNA polymerase (*Replinase* from Dupont (UK) Ltd), 200µM dNTPs in a total volume of 100 µl buffer (50 mM Tris pH 9, 20 mM ammonium sulphate, 1.5 mM $MgCl_2$). Thirty to 50 cycles of amplification were performed in Cetus *Thermal Cycler*. Essentially, DNA amplification was performed with a pair of outboard primers around either exon 5, exon 7 or exon 8, and then the amplified fragment purified by excision of the specific band from a 2.5% agarose gel (2% Nusieve, 0.5% Seakem) and recovery of the DNA by electrophoretic concentration (*Extraphor Electrophoretic Concentrator*, LKB Pharmacia). 10 ng of template DNA was mixed with 20 pmol of relevant inboard primer in a final volume of 8 µl and denatured by heating to 98°C for 7 min, and then 2 µl of 5 × Sequenase reaction buffer (United States Biochemical) added. The mixture was then incubated at 37°C for 30 min, then at room temperature for 10 min and finally on ice before proceeding with the standard Sequenase Version 2.0 (United States Biochemical) sequencing protocol using $^{35}$S-dATP.

### Results

### *Nuclear immunoreactivity*

Of the 22 primary exocrine pancreatic carcinomas studied by immunohistochemistry in frozen sections, 13 (60%) possessed nuclear immunoreactivity with the JG8 and CM1 polyclonal antisera and of these 11 had immunoreactivity with the mutant-specific monoclonal antibody 240 (PAb240). Not all malignant cells within an individual tumour were positive, and the percentage of cells showing definite immunoreactivity with either antibody varied from 20% up to 90%. Cases in

**Figure 1** Regions of p53 gene exons 5, 7 and 8 analysed in genomic DNA extracted from archival pathology specimens. The conserved regions II, III, IV and V are indicated in the exons 5 through 8, and the numbered arrowheads represent the position of the primers used for DNA amplification and sequencing.

which there was faint cytoplasmic staining without nuclear staining were not scored as positive in this analysis.

Seven of the 13 cell lines examined by immunocytochemistry on cells grown on glass slides showed nuclear immunoreactivity with PAb240, PAb421, PAb1801 and the polyclonal antiserum CM1 (Table I); six of these cell lines also showed reactivity with the antiserum JG8. There was some variation in the preparation of cells positive with each test (Table I).

The polyclonal antiserum CM1 was used to detect p53 immunoreactivity in 124 pancreatic carcinoma cases in routinely-processed paraffin sections. Twenty-eight cases (23%) were positive for nuclear immunoreactivity with this antiserum (Figure 2). The percentage of positive tumours did not vary significantly with pathological grade (Table II). In some cases there was definite cytoplasmic staining in addition to convincing nuclear positivity. We did not observe this phenomenon in frozen sections tested with this antibody, and it may represent an effect of fixation and processing. Five cases of intraductal carcinoma (which is thought to represent an earlier stage of tumorigenesis than invasive carcinoma, analogous to the situation in breast cancer) were examined with the CM1 antiserum, and two of these showed nuclear immunoreactivity in tumour cells.

**Table II** Abnormalities of p53 expression in paraffin-embedded cases of pancreatic cancer detected with CM1 antiserum

| *Tumour grade*[a] | *1* | *2* | *3* |
|---|---|---|---|
| CM1 immunoreactive/total | 11/52 | 12/48 | 5/24 |
| % Immunoreactive | 21% | 25% | 21% |

[a]Grade as defined by Kloppel and Maillet (1989), with grade 1 tumours having lowest mitotic activity and best differentiation.

### Immunoprecipitation studies

Thirteen pancreatic cancer cell lines were examined in immunoprecipitation assays with the monoclonal antibodies

**Table I** Abnormalities of p53 in human pancreatic carcinoma cell lines

| | *Immunocytochemistry*[a] | | | | | *Immuno-precipitation*[b] | | *Sequence analysis* |
|---|---|---|---|---|---|---|---|---|
| | *240* | *1801* | *421* | *JG8* | *CM1* | *240* | *1801* | |
| Panc1 | ++++ | ++++ | ++++ | +++ | ++++ | ++ | ++++ | $CGT^{273} \rightarrow CAT^{273}$ |
| MiaPaCa2 | ++++ | ++++ | ++++ | ++++ | ++++ | +++ | ++++ | $CGG^{248} \rightarrow TGG^{248}$ |
| PT45 | ++++ | ++++ | ++++ | +++ | ++++ | ++ | ++++ | $AGA^{280} \rightarrow AAA^{280}$ |
| HPAF | + | + | + | + | + | +/− | + | $CCC^{151} \rightarrow CTC^{151}$ |
| PSN1 | ++ | ++ | ++ | ++ | ++ | + | + | $AAG^{132} \rightarrow CAG^{132}$ |
| CaPan1 | ++ | ++++ | +++ | + | ++ | − | − | None detected |
| CaPan2 | − | − | − | − | − | − | − | None detected |
| CaP1M | − | − | − | − | − | − | − | None detected |
| AsPc1 | − | − | − | − | − | − | − | None detected |
| BxPc3 | + | + | + | − | + | − | − | None detected |
| 818.1 | − | − | − | − | − | − | − | None detected |
| 818.4 | − | − | − | − | − | − | − | None detected |
| Colo357 | − | − | − | − | − | − | − | None detected |

[a]Positive signals were graded for proportion of cells with nuclear staining (+ <25%, ++ 25–50%, +++ 50–75%, ++++ 75–100%). [b]Positive signals were graded for intensity (+ to ++++).

**Figure 2** Immunoreactivity for p53 detected with the CM1 antiserum in a formalin-fixed, paraffin-embedded pancreatic adenocarcinoma grade 2 showing typical perineural invasion. The tumour cells show dense nuclear immunoreactivity while the stromal cells are negative (× 312, haematoxylin counterstain).

PAb1801 and 240. Five cell lines had detectable p53 (Figure 3) on immunoprecipitation with antibody PAb1801, and four of these also showed a band of lesser intensity on immunoprecipitation with the mutant-specific antibody PAb240 (Table I).

*Point mutations of p53 gene*

Several studies have established that immunoreactivity with monoclonal anti-p53 antibodies PAb421, 240 and 1801 is associated with point mutations of the p53 gene (Iggo *et al.*, 1990; Rodrigues *et al.*, 1990). All of the previous studies have been restricted to frozen tumour samples or cell lines because the monoclonal antibodies available do not recognise p53 in formalin-fixed material, and the techniques developed to identify point mutations have been designed for use on cDNA or high molecular weight genomic DNA. We have developed a sequencing strategy (Wright *et al.*, 1991) that can be applied to the low molecular weight genomic DNA obtainable from fixed tissue sections, amplifying the most conserved parts of p53 in exons 5, 7 and 8 representing the 'hot spots' for mutation (Baker *et al.*, 1990) in box II (codon 132–145), box III (codon 171–179), box IV (codon 239–248) and box V (codon 272–286).

To examine the relationship between immunoreactivity with the CM1 antiserum in formalin-fixed, paraffin-embedded material and p53 point mutations, we attempted amplification and sequence analysis in eight tumours with positive nuclear staining and four tumours without staining. We failed to amplify specific DNA in three of the CM1-positive cases and in one of the CM1-negative cases. We found point mutations in three of the CM1-positive cases (codon 246 ATG to ATT, methionine to isoleucine; codon 249 AGG to AAG, arginine to leucine; codon 273 CGT to CAT, arginine to histidine), confirmed on both sense and antisense strands. We infer that the mutant gene is hemizygous in each of these cases because the band representing wild type sequence is absent or much weaker than the band for the mutant sequence (Figure 4). We do not have high molecular weight DNA available for these cases and so are unable to confirm allelic deletions by RFLP analysis of chromosome 17p. Only wild type sequence was present in the CM1-negative cases. In this small series point mutations in the hot spots were therefore restricted to CM1-positive cases, although of course we have not excluded abnormalities in other areas of the p53 gene.

We used direct sequencing of product amplified from genomic DNA and cDNA of each of the cell lines to confirm expression of a mutant p53 gene. Point mutations of the coding sequence in exons 5, 6, 7 and 8 were identified (Figures 5 and 6) in all of the cell lines overexpressing p53 by immunoprecipitation analysis (Panc-1, HPAF, PT45, MiaPaCa-2 and PSN-1). The CaPan1 and BxPc3 cell lines were positive by immunocytochemistry but negative by immunoprecipitation analysis, and no mutation in any exon was identified in these lines. Since p53-immunoreactive cells form only a minority population in the BxPc3 cell line it is possible that we could miss a mutation by the direct sequencing technique, particularly if the mutation is heterozygous. The explanation for our failure to identify an activating mutation in the CaPan1 cell line is currently obscure, but it is interesting that immunoprecipitation analysis failed to detect high levels of mutant p53 in contrast to the immunocytochemical analysis. None of the cell lines that lacked immunoreactive p53 protein possessed point mutations in any exon.

### Discussion

Our results indicate that p53 abnormalities occur at high frequency in pancreatic cancer, similar to the frequencies reported in other common human malignancies such as colorectal cancer (Rodrigues *et al.*, 1990; Baker *et al.*, 1990), breast cancer (Bartek *et al.*, 1990), lung cancer (Chiba *et al.*, 1990) and hepatocellular cancer (Hsu *et al.*, 1991; Bressac *et al.*, 1991).

Using frozen tissue and a panel of anti-p53 antibodies (including the CM1 polyclonal antiserum) we detected overexpression of mutant p53 protein in 60% of cases of pancreatic cancer. With paraffin sections, using only the CM1 antiserum, we found a smaller proportion of cases (20–25%) showing nuclear immunoreactivity. This difference in p53 immunoreactivity is probably because the epitope recognised by CM1 antiserum is only partially resistant to formalin fixation. Examination of genomic DNA extracted from representative paraffin sections confirmed that point mutations were present in conserved regions of the p53 gene in those cases showing positive p53 immunoreactivity.

Sequence analysis of those cases expressing high levels of p53 protein also allowed identification of a point mutation in the coding sequence of exons 5, 6 7 or 8 in most cases. The nature of the base change involved showed no special predilection, with G to A transitions, C to T transitions and G to T transversions identified in this series. In non-small cell

**Figure 3** Immunoblot of immunoprecipitates from pancreatic cancer cell lines. Extracts were immunoprecipitated with PAb240 or PAb1801 for each cell line. Immunoblots were probed with rabbit anti-p53 serum JG8 at 1/100 dilution.

**Figure 4** Sequencing of p53 in genomic DNA extracted from archival pathology specimens. Two cases, lanes 2 and 4, show mutations in the coding sequence in conserved region IV.

**Figure 5** Sequencing of p53 in genomic DNA from pancreatic cell lines. The MiaPaCa-2 cell line shows a mutation at codon 248 (CGG to TGG, amino acid arginine to tryptophan) in conserved region IV.

**Figure 6** Sequencing of p53 in genomic DNA from pancreatic cell line PT45 shows a mutation at codon 280 (AGA to AAA, amino acid arginine to lysine) in conserved region V.

lung cancer (Chiba *et al.*, 1990) and hepatocellular cancers (Hsu *et al.*, 1991; Bressac *et al.*, 1991) there is a very high frequency of G to T transversions, while in most other tumour types there is an excess of G to A transitions (Baker *et al.*, 1989; Bartek *et al.*, 1990; Nigro *et al.*, 1990). These differences have been interpreted as evidence of the involvement of specific mutagenic agents in the various types. In pancreatic cancer there is a strikingly high frequency of point mutation at codon 12 of the Ki-*ras* oncogene. While there is absolute restriction to this single codon of this single member of the *ras* family, there is no particular base substitution. G to A transitions and G to T transversions are identified in about the same number of cases and G to C transversions rather less commonly (Bos *et al.*, 1989). Therefore we have little evidence to indicate the action of a single mutagen in the genesis of pancreatic cancer and this is consistent with the relative lack of epidemiological evidence implicating environmental agents in pancreatic carcinogenesis. Only a relatively weak association with environmental factors such as cigarette smoking, alcohol and coffee drinking have been described (Doll & Peto, 1976; McMahon, 1982). In contrast, both hepatocellular cancer and non-small cell lung cancer have relatively strong associations with potent environmental carcinogens: hepatocellular cancer with hepatitis B virus and aflatoxins, and lung cancer with cigarette smoking.

The molecular basis of pancreatic cancer is now becoming clearer, and a number of independent genetic events have been identified in human tumours (Lemoine & Hall, 1990). In addition to a very high frequency of Ki-*ras* activation by point mutation (Shibata *et al.*, 1990), there is evidence of a autocrine loop involving overexpression of transforming growth factor alpha and the epidermal growth factor receptor (Barton *et al.*, 1991; Lemoine *et al.*, 1991*a*). These abnormalities may be linked for there is *in vitro* evidence that activation of *ras* oncogenes causes upregulation of TGF alpha expression (Ciardello *et al.*, 1990). Overexpression (sometimes with amplification) of the c-*erb*B-2 proto-oncogene has been described in about 20% of pancreatic cancers (Hall *et al.*, 1990). Now we have demonstrated inactivation of the p53 tumour suppressor gene in a large proportion of cases.

The relative importance and sequence of these genetic events in pancreatic carcinogenesis remains to be determined, although we do know that both overexpression of mutant p53 and point mutation of Ki-*ras* are found in intraductal carcinoma *in situ* lesions (this study and Lemoine *et al.*, 1991*b*). *In vitro*, normal human epithelial cells may be partially transformed by activated *ras* oncogenes alone, but for full malignant transformation an additional genetic event, associated with immortalisation, is necessary (Burns *et al.*, 1991). This additional genetic event may involve the loss or inactivation of a tumour suppressor gene. In patients with Li-Fraumeni syndrome, characterised by a hereditary predisposition to various tumours including pancreatic cancer, germ-line mutations of the p53 gene have been described (Malkin *et al.*, 1990; Srivastava *et al.*, 1990). In these patients, p53 abnormalities are clearly the earliest genetic event in tumorigenesis. In sporadic colorectal cancer, however, p53 mutation appears to be a late event and it has been suggested that the order of events is not important, the net result being dependent on the accumulation of genetic abnormalities (Baker *et al.*, 1990; Vogelstein, 1991). In the pancreas carcinoma *in situ* and other lesions interpreted as intermediate in the progression to invasive cancer are very rarely reported by pathologists, and so it may be difficult to pinpoint the appearance of p53 in the temporal sequence of this tumour.

The authors are grateful to the Imperial Cancer Research Fund, Mike Stone Cancer Research Fund Glaxo plc and the Cancer Research Campaign for financial support. We thank Ian Goldsmith for production of oligonucleotides and Arthur McKie for expert technical assistance.

### References

BAKER, S.J., PREISINGER, A.C., JESSUP, J.M. & 5 others (1990). P53 gene mutations occur in combination with 17p allelic deletions as late events in colorectal tumorigenesis. *Cancer Res.*, **50**, 7717.

BANKS, L., MATLASHEWSKI, G. & CRAWFORD, L. (1986). Isolation of human p53-specific monoclonal antibodies and their use in the studies of human p53 expression. *Eur. J. Biochem.*, **159**, 529.

BARTEK, J., IGGO, R., GANNON, J. & LANE, D.P. (1990). Genetic and immunochemical analysis of mutant p53 in human breast cancer cell lines. *Oncogene*, **5**, 893.

BARTEK, J., BARTKOVA, J., VOJTESEK, B. & 7 others (1991). Aberrant expression of the p53 oncoprotein is a common feature of a wide spectrum of human malignancies. *Oncogene*, **6**, 1699.

BARTON, C.M., HALL, P.A., HUGHES, C.M., GULLICK, W.J. & LEMOINE, N.R. (1991). Transforming growth factor alpha and epidermal growth factor in human pancreatic cancer. *J. Pathol.*, **163**, 111.

BOS, J.L. (1989). *Ras* oncogenes in human cancer. *Cancer Res.*, **49**, 4682.

BRESSAC, B., KEW, M., WANDS, J. & OZTURK, M. (1991). Selective G to T mutations of p53 gene in hepatocelular carcinoma from Southern Africa. *Nature*, **350**, 429.

BUCHMAN, V.L., CHUMAKOV, P.M., NINKINA, N.N., SAMARINA, O.P. & GEORGIEV, G.P. (1988). A variation in the structure of the protein-coding region of the human p53 gene. *Gene*, **70**, 245.

BURNS, J.S., BARTON, C.M., WYNFORD-THOMAS, D. & LEMOINE, N.R. (1992). *In vitro* transformation of epithelial cell by *ras* oncogenes. *Epithelial Cell Biol.* (in press).

CANCER OF THE PANCREAS TASK FORCE GROUP (1981). Staging of cancer of the pancreas. *Cancer*, **47**, 1631.

CHIBA, I., TAKAHASHI, T., NAU, M.M. & 11 others (1990). Mutations in the p53 gene are frequent in primary resected non-small cell lung cancer. *Oncogene*, **5**, 1603.

CHUMAKOV, P.M., ALMAZOV, V.P. & JENKINS, J.R. (1990). The complete nucleotide sequence of the human p53 gene. *Proceedings of the Fourth International p53 Workshop*, Oxted: UK.

CIARDELLO, F., MCGEADY, M.L., KIM, M. & 11 others (1990). Transforming growth factor alpha is enhanced in human mammary epithelial cells transformed by an activated c-Ha-*ras* proto-oncogene but not by the c-*neu* proto-oncogene, and overexpression of the transforming growth factor alpha complementary DNA leads to transformation. *Cell Growth & Differentiation*, **1**, 407.

DOLL, R. & PETO, R. (1976). Mortality in relation to smoking: 20 years observations on male British doctors. *Br. Med. J.*, **2**, 1525.

GANNON, J.V., GREAVES, R., IGGO, R. & LANE, D.P. (1990). Activating mutations in p53 produce common conformational effects. A monoclonal antibody specific for the mutant form. *EMBO J.*, **9**, 1595.

HALL, P.A., HUGHES, C.M., STADDON, S.L., RICHMAN, P.I., GULLICK, W.J. & LEMOINE, N.R. (1990). The c-*erb*B-2 proto-oncogene in human pancreatic cancer. *J. Pathol.*, **161**, 195.

HARLOW, E. & LANE, D.P. (1988). *Antibodies: A Laboratory Manual.* Cold Spring Harbor Laboratory Press: New York.

HIGUCHI, R. (1989). Simple and rapid preparation of samples for PCR. In Erlich, H.A. (ed.). *PCR Technology: Principles and Applications for DNA Amplification*, pp. 31–38, Stockton Press: New York.

HINDS, P.W., FINLAY, C.A., QUARTIN, R.S. & 4 others (1990). Mutant p53 DNA clones from human colon carcinomas cooperate with *ras* in transforming primary rat cells: a comparison of the 'hot spot' mutant phenotypes. *Cell Growth & Differentiation*, **1**, 571.

HSU, I.C., METCALF, R.A., SUN, T., WELSH, J.A., WANG, N.J. & HARRIS, C.C. (1991). Mutational hotspots in the p53 gene in human hepatocellular carcinomas. *Nature*, **350**, 427.

IGGO, R., GATTER, K.,M BARTEK, J., LANE, D.P. & HARRIS, A.L. (1990). Increased expression of mutant forms of p53 oncogene in primary lung cancer. *Lancet*, **335**, 675.

KLÖPPEL, G. & MAILLET, B. (1989). Classification and staging of pancreatic nonendocrine tumors. *Radiological Clinics of North America*, **27**, 105.

LEMOINE, N.R. & HALL, P.A. (1990). Growth factors and oncogenes in pancreatic cancer. *Baillière's Clinical Gastroenterol.*, **4**, 815.

LEMOINE, N.R., MAYALL, E.S., WYLLIE, F.A. & 4 others (1989). High frequency of ras oncogene activation in all stages of human thyroid tumorigenesis. *Oncogene*, **4**, 159.

LEMOINE, N.R., HUGHES, C.M., BARTON, C.M. & 6 others (1991*a*). The epidermal growth factor receptor in human pancreatic cancer. *J. Pathol.* (in press).

LEMOINE, N.R., JAIN, S., HUGHES, C.M. & 4 others (1991*b*). Ki-*ras* oncogene activation in invasive ductal adenocarcinoma, but not in ductal papillary hyperplasia or intraductal papillary neoplasia of the pancreas. *Gastroenterology*, **101**, 1.

MALKIN, D., LI, F.P., STRONG, L.C. & others (1990). Germ-line p53 mutations in a familial syndrome of breast cancer, sarcomas, and other neoplasms. *Science*, **250**, 1233.

MCMAHON, B. (1982). Risk factors for cancer of the pancreas. *Cancer*, **50**, 2676.

NIGRO, J.M., BAKER, S.J., PREISINGER A.C. & 13 others (1989). Mutations in the p53 gene occur in diverse human tumour types. *Nature*, **342**, 705.

RODRIGUES, N.R., ROWAN, A., SMITH, M.E. & 4 others (1990). P53 mutations in colorectal cancer. *Proc. Natl Acad. Sci. USA*, **87**, 7555.

SHIBATA, D., CAPELLA, G. & PERUCHO, M. (1990). Mutational activation of the c-K-*ras* gene in human pancreatic carcinoma. *Ballière's Clinical Gastroenterology*, **4**, 151.

SRIVASTAVA, S., ZOU, Z., PIROLLO, K., BLATTNER, W. & CHANG, E.H. (1990). Germ-line transmission of a mutated p53 gene in a cancer-prone family with Li-Fraumeni syndrome. *Nature*, **248**, 747.

TAKAHASHI, T., NAU, M.M., CHIBA, I. & 7 others (1989). P53: a frequent target for genetic abnormalities in lung cancer. *Science*, **246**, 491.

VOGELSTEIN, B. (1991). A deadly inheritance. *Nature*, **348**, 681.

WADE-EVANS, A. & JENKINS, J.R. (1985). Precise epitope mapping of the murine transformation-associated protein, p53. *EMBO J.*, **4**, 699.

WILLIAMSON, R.C.N. (1988). Pancreatic cancer: the greatest oncological challenge. *Br. Med. J.*, **296**, 445.

WRIGHT, P.A., LEMOINE, N.R., GORETSKI, P.E. & 6 others (1991). Mutation of the p53 gene in a differented human thyroid carcinoma cell line, but not in primary thyroid tumours. *Oncogene*, **6**, 1693.