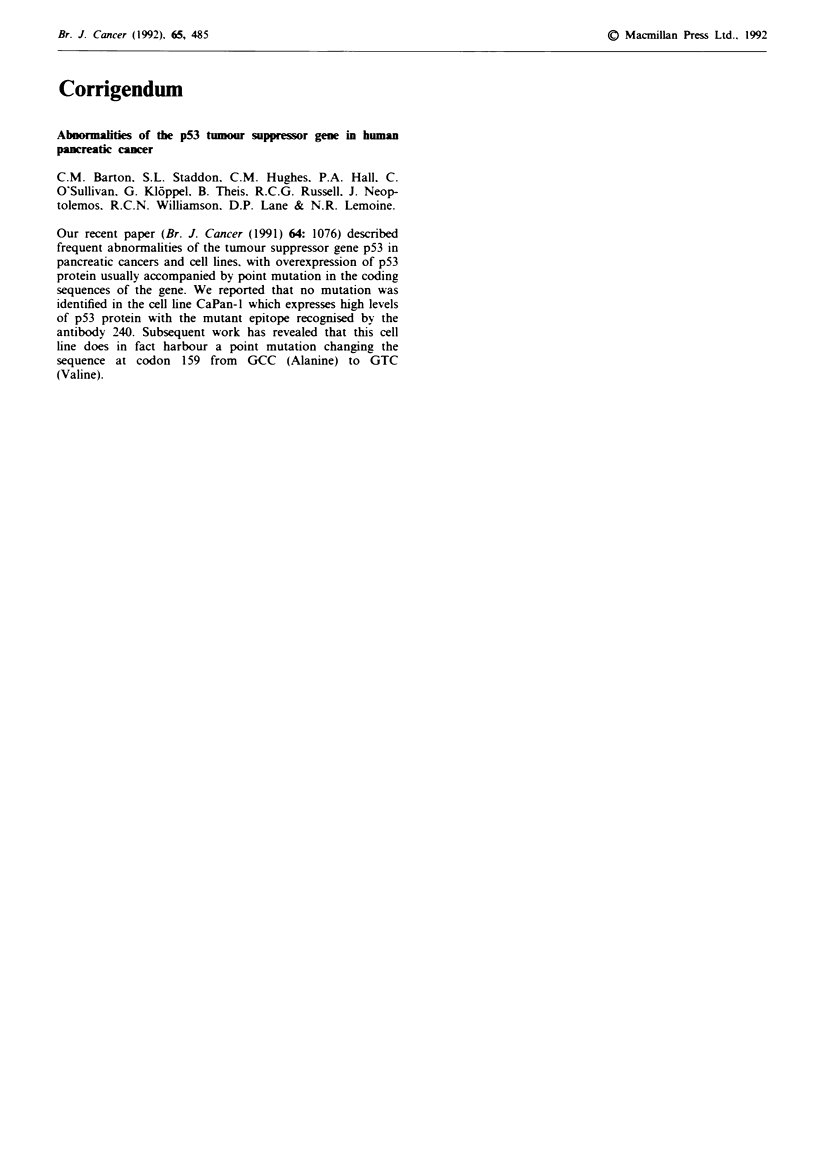# Abnormalities of the p53 tumour suppressor gene in human pancreatic cancer

**Published:** 1992-03

**Authors:** 


*Br. J. Cancer* (1992), **65**, 485 © Macmillan Press Ltd., 1992

### Corrigendum

**Abnormalities of the p53 tumour suppressor gene in human pancreatic cancer**

C.M. Barton, S.L. Staddon, C.M. Hughes, P.A. Hall, C. O'Sullivan, G. Klöppel, B. Theis, R.C.G. Russell, J. Neoptolemos, R.C.N. Williamson, D.P. Lane & N.R. Lemoine.

Our recent paper (*Br. J. Cancer* (1991) **64**: 1076) described frequent abnormalities of the tumour suppressor gene p53 in pancreatic cancers and cell lines, with overexpression of p53 protein usually accompanied by point mutation in the coding sequences of the gene. We reported that no mutation was identified in the cell line CaPan-1 which expresses high levels of p53 protein with the mutant epitope recognised by the antibody 240. Subsequent work has revealed that this cell line does in fact harbour a point mutation changing the sequence at codon 159 from GCC (Alanine) to GTC (Valine).